# Intratumoral injection therapies for locally advanced pancreatic cancer: systematic review

**DOI:** 10.1093/bjsopen/zrad052

**Published:** 2023-05-31

**Authors:** Coen Ysbrand Willink, Sjoerd Franciscus Maria Jenniskens, Nienke Johanna Maria Klaassen, Martijn Willem Jan Stommel, Johannes Frank Wilhelmus Nijsen

**Affiliations:** Department of Medical Imaging, Radboud Institute for Health Sciences, Radboud University Medical Centre, Nijmegen, The Netherlands; Department of Medical Imaging, Radboud Institute for Health Sciences, Radboud University Medical Centre, Nijmegen, The Netherlands; Department of Medical Imaging, Radboud Institute for Health Sciences, Radboud University Medical Centre, Nijmegen, The Netherlands; Department of Surgery, Radboud Institute for Health Sciences, Radboud University Medical Centre, Nijmegen, The Netherlands; Department of Medical Imaging, Radboud Institute for Health Sciences, Radboud University Medical Centre, Nijmegen, The Netherlands

## Abstract

**Introduction:**

Pancreatic cancer has one of the worst prognoses of all cancers. Patients with locally advanced pancreatic cancer have a 12.7–20.2 per cent chance of receiving curative surgery after induction systemic chemotherapy. Intratumoral injection therapies have been studied as complementary treatment options for improved local tumour control. The aim of this systematic review was to provide an overview of intratumoral injection therapies, their safety, and oncological outcome in patients with locally advanced pancreatic cancer.

**Methods:**

A literature search was conducted in PubMed, Embase and the Cochrane Library for articles written in English up to 28 November 2022. All study designs involving at least five patients with locally advanced pancreatic cancer who were treated with an intratumoral injection therapy were included. Critical appraisal of the included studies was performed using the Newcastle–Ottawa scale.

**Results:**

After evaluation of the 1680 articles yielded by the systematic search, 52 studies treating 1843 patients were included. Included intratumoral injection treatment modalities comprised iodine-125 (^125^I) seed brachytherapy (32 studies, 1283 patients), phosphorus-32 (^32^P) microbrachytherapy (5 studies, 133 patients), palladium-103 (^103^Pd) seed brachytherapy (2 studies, 26 patients), immunotherapy (9 studies, 330 patients), and chemotherapy (4 studies, 71 patients). Overall survival ranged between 7.0 and 16.0 months for ^125^I, 5.2 and 15.5 months for ^32^P, 6.9 and 10.0 months for ^103^Pd, 5.8 and 13.8 months for immunotherapy, and 9.0 and 16.2 months for chemotherapy. Severe complication (greater than or equal to grade III complications using Clavien–Dindo classification) rates were 6.2 per cent for ^125^I, 49.2 per cent for ^32^P, 15 per cent for ^103^Pd, 57.9 per cent for immunotherapy, and 0 per cent for chemotherapy.

**Conclusion:**

Five intratumoral injection therapies are described and an overview is reported. Some intratumoral injection therapies for patients with locally advanced pancreatic cancer seem safe, although ^32^P microbrachytherapy and immunotherapy require additional evidence. Currently available data are insufficient to provide firm conclusions regarding the added value to survival. The potential advantage of intratumoral injection therapies complementary to conventional care should be studied in well designed RCTs.

## Teaser

Intratumoral injection therapies have been studied as novel (complementary) treatment options for improved local tumour control of locally advanced pancreatic cancer. A literature search was conducted in PubMed, Embase, and the Cochrane Library, including 52 studies treating 1843 patients with 5 different modalities of intratumoral injection therapies. There was a large variation in safety and survival; however, most intratumoral injection therapies seem safe, although current data are insufficient for firm conclusions regarding the added value to survival.

## Introduction

Pancreatic cancer is diagnosed in over 440 000 people worldwide every year and the incidence has increased by 55 per cent over the past 25 years^1^. The mortality is similar to the incidence due to the poor prognosis of this malignancy. With a 1-year overall survival (OS) of just 20 per cent and 5-year survival of 9 per cent, pancreatic cancer is one of the most aggressive forms of all common cancers^2^. When untreated, 5-year survival decreases to 3 per cent^3^. Resection can be performed in just 20 per cent of all patients and is the only potentially curative treatment option. At the time of diagnosis, around 50 per cent of all patients with pancreatic cancer are affected by distant metastases and the remaining 30 per cent have locally advanced pancreatic cancer (LAPC), making resection futile^4,5^. The most commonly used criteria for LAPC are those from the National Comprehensive Cancer Network (NCCN) guidelines, defining LAPC as greater than 180° arterial encasement or unreconstructible venous involvement with­out evidence of distant metastases^6^. Commonly, tumour involvement in the superior mesenteric artery, celiac axis, or common hepatic artery or definite occlusion of the superior mesenteric vein or portal vein make pancreatic cancer unresectable^7^.

The current therapy of choice for LAPC is induction/palliative chemotherapy with FOLFIRINOX (a combination of 5-fluorouracil, irinotecan, leucovorin, and oxaliplatin) or gemcitabine with nab-paclitaxel, and response evaluation after 8 weeks^8^. During re-evaluation, if metastases remain absent and no tumour progression is observed, approximately 28.0–31.3 per cent become eligible for exploration surgery, 12.6–20.2 per cent receive surgical resection, and 15.9–18.1 per cent have an R0 (greater than 1 mm) outcome^9,10^. Of the 79.8–87.3 per cent of patients that do not receive surgical resection, approximately 25.0 per cent have local tumour progression without metastatic disease and may benefit from local therapies^9,10^. Gemcitabine has been the recommended induction therapy for LAPC for over a decade and is still used in patients with a WHO performance score of 2 and higher^8^. OS for LAPC is approximately 14.8–24.2 months for FOLFIRINOX chemotherapy^11,12^ and 9.0–16.0 months for gemcitabine-based chemotherapy^9,13^; however, most patients also undergo radiotherapy, ablation therapies, second-line chemotherapy, or resection before, during, or after the first-line chemotherapy treatment. For patients with severe co-morbidities these extensive combination treatments are often considered impossible^14^. Some studies suggest that almost half of elderly patients (greater than 65 years) with no metastatic pancreatic cancer do not undergo chemotherapy or surgery, possibly due to co-morbidities^15^.

For patients with stable unresectable disease after chemotherapy, local ablation is occasionally applied in clinical trials aiming to control local progression and to prolong survival^16,17^. Although ablation is considered feasible, it is also associated with substantial morbidity and mortality^16^. The effectiveness of additional local ablation is disputable because of the paucity of high-level evidence. Overall, small non-comparing case studies, hampered by selection bias, show a wide variation in OS from 5.0 up to 25.6 months^16^. Although some survival outcomes after ablation seem promising, the clinical demand for a minimally invasive therapy to improve local tumour control is still unmet.

Optimally, a local therapy for pancreatic cancer is minimally invasive, offers accurate treatment delivery with complete tumour coverage, spares healthy surrounding tissue, and has accurate therapy prediction and control. To meet these demands, over the past decades, novel intratumoral injection therapies for pancreatic cancer have been studied worldwide. Advancements in therapy control, advanced image acquisition and processing, personalized treatment planning and immunological pathways have changed the perspective to achieve optimal local tumour control. With less invasive therapies, hospital stay and healthcare costs may decrease^18^. Safe treatment delivery reduces complication rates and benefits the quality of life. The aim of this systematic review was to provide an overview of intratumoral injection therapies, their safety, and oncological outcome in patients with LAPC.

## Methods

This systematic review was conducted and reported conforming to PRISMA guidelines^19^. The methodology and inclusion/exclusion criteria were defined in advance by a Biomedical Information Specialist and the authors. This study was registered on PROSPERO, the international prospec­tive register of systematic reviews (registration ID: CRD42020212862).

### Search strategy

A literature search was conducted in PubMed, Embase, and the Cochrane Library for articles written in English from dates of inception up to 28 November 2022. The literature search was performed using medical domains combined by ‘AND’ between domains and within the domain by ‘OR’. The first domain contained terms regarding pancreatic cancer, the second regarding intratumoral therapy, and the third regarding LAPC. Search terms were restricted to Medical Subject Headings, title, abstract, and keywords. Study selection and organization were performed using EndNote X9.2. The complete search strategy for each library is presented in *Appendix S1*. After a first scan to remove duplicate publications, the titles and abstracts were scanned for inclusion and exclusion criteria. Publications limited to an abstract were not excluded if the information was adequate, as described below. If multiple studies contained the same patient cohort, only the latest published article was included. If there was uncertainty regarding inclusion, a second author was consulted.

### Definitions

LAPC was defined as an irresectable tumour due to vascular involvement without distant metastasis. Patients with vascular involvement resected at diagnosis or at any time during follow-up (for example after induction chemotherapy) were not considered. A more detailed definition of LAPC (for example type and extent of vascular involvement)^20^ could not be applied due to the time span and heterogeneity of the included studies.

Intratumoral injection therapy was defined as the injection of an active substance in the pancreatic tumour mass with the intention to treat or control the primary pancreatic cancer. Angiographically delivered therapy, infusion therapy, stent­ing, ablation, or post-resection treat­ments were not defined as intratumoral injection therapy. Studies performing non-resection surgical procedures, including cholangiojejunostomy, gastro­jejunos­tomy, biliary/gastric bypass, and stent placement, were included if performed complementary or secondary to intratumoral injection therapy.

### Study selection

Studies were included if they treated human patients suffering from LAPC with a single intratumoral injection therapy and presented outcomes regarding survival and/or safety. Articles had to be published in a registered journal defined by the SCImago Journal & Country Rank^21^. Studies were excluded if one or more of the following criteria was met: reviews, non-English articles, animal studies, case studies (or less than five patients in a single treatment population), minority LAPC in a treatment population, and resection immediately after intratumoral injection therapy.

### Quality assessment

All studies passing the full-text assessment were critically appraised according to the Newcastle–Ottawa scale (NOS) for assessing the quality of non-randomized studies. The NOS is a validated scoring system with appraisals for case–control and cohort studies. RCTs were assessed using the cohort evaluation. A total of nine points could be appraised per study; four by selection, two by comparability, and the last three by either exposure or outcome of interest for case–control and cohort studies respectively. The complete scoring criteria are presented in *Appendix S2*. Studies with five stars or more were considered of good quality. Studies with less than five stars were not excluded.

### Data extraction

Data on intratumoral injection therapy, dose, approach, cancer stage, metastases, combination therapies, median OS, and complications by the Clavien–Dindo classification^22^ were extracted when available^23^. Furthermore, study characteristics, such as design, country, population characteristics, and sample size, were extracted from the included studies. Data extraction and organization were performed using Microsoft^®^ Excel^®^ for Microsoft 365.

### Statistical analysis

Most outcomes were descriptive and, due to the heterogeneity of the included studies, no meta-analysis or statistical analysis was performed.

## Results

**Fig. 1:**
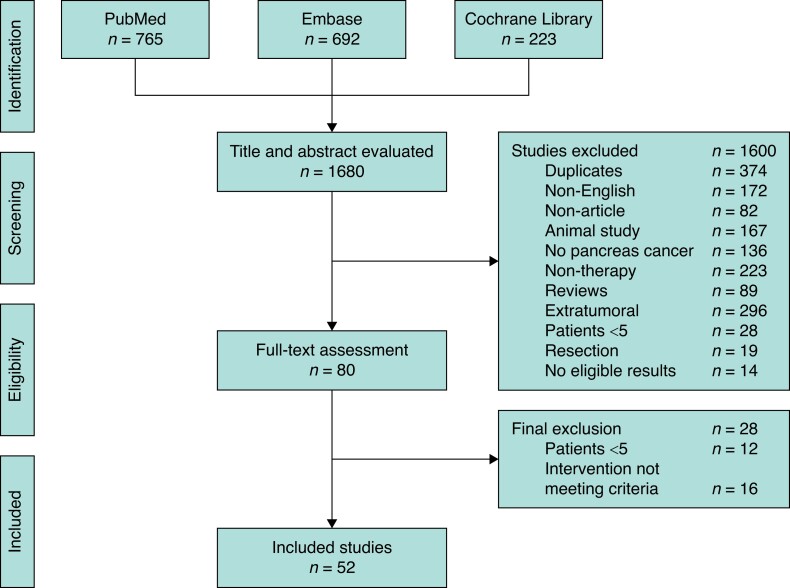
PRISMA flow chart of the study selection

Starting with 1680 articles, after title and abstract screening for duplicates and exclusion criteria, 1600 studies were excluded. Eighty studies entered full-text assessment. Of these, 28 studies were excluded because of small sample size (12) and/or intervention not meeting the inclusion criteria (16). Some 52 clinical studies with 1843 patients were included for quality assessment. The complete results of the quality assessment are reported in *Appendix S3*. A detailed selection flow chart is shown in *Fig. 1*.

The included studies comprised five different intratumoral injection treatment modalities: iodine-125 (^125^I) seed brachytherapy (32 studies, 1283 patients), phosphorus-32 (^32^P) microbrachytherapy (5 studies, 133 patients), palladium-103 (^103^Pd) seed brachytherapy (2 studies, 26 patients), immunotherapy (9 studies, 330 patients), and chemotherapy (4 studies, 71 patients). Most of the included studies had the following inclusion criteria in common: age greater than or equal to 18 years, adequate performance status (WHO/Eastern Cooperative Oncology Group (ECOG), Karnofsky), and an adequate hepatic, haematological, immune, and/or renal function. Gemcitabine-based chemotherapy was the most frequently used form of induction/palliative chemotherapy. One study combined intratumoral injection therapy with FOLFIRINOX chemotherapy^24^. All results are presented per modality and an overview of all intratumoral injection therapies is presented in *Table 1*.

**Table 1:** Results of all intratumoral injection therapies for locally advanced pancreatic cancer

Intratumoral injection therapy	No. of studies	No. of patients	Metastasis	Chemotherapy	Chemoradiotherapy	Radiotherapy	Overall complications, *n*	Complications ≥grade III	OS range (months)
Iodine-125	32	1283	212 of 1026 (20.6)	538 (47.5)	161 (14.2)	137 (12.1)	324 in 771	37 (6.2)	7–16
Phosphorus-32	5	133	19 of 133 (14.3)	53 (39.8)	65 (48.9)	0	1122 in 89	61 (49.2)	5.2–15.5
Palladium-103	2	26	1 of 26 (4)	2 (8)	20 (77)	0	6 in 26	4 (15)	5–7
Immunotherapy	9	330	147 of 294 (50.0)	72 (21.8)	252 (76.4)	0	316 in 223	33 (57.9)	5.8–13.8
Chemotherapy	4	71	15 of 53 (28)	18 (33)	36 (67)	0	3 in 41	0 (0)	9–16.2

Values are *n* (%) unless otherwise stated. OS, overall survival.

### Iodine-125 seed brachytherapy

An overview of the results of intratumoral injection ^125^I brachytherapy is presented in *Table 2*. Of the 32 studies applying ^125^I brachytherapy in 1283 patients suffering from pancreatic adenocarcinoma, 15 had a retrospective design^25–39^, 16 had an open-label prospective design^40–55^, and one compared ^125^I combined with chemotherapy *versus* chemotherapy alone in an RCT^56^. An overview of the characteristics of all applied radioactive isotopes is presented in *Appendix S4*.

**Table 2:** Results of intratumoral iodine-125 seed brachytherapy for locally advanced pancreatic cancer

Reference	*n*	Metastasis	Tumour dose (Gy), median* (range)	Combination therapy	Overall complications, *n*	Complications ≥grade III	Median* OS (months); OS initiation	NOS 1–9
**Intraoperative**								
Dobelbower *et al*., 1986^40^	12	2 (17)	140 (120–210)	CRT 6 (50)	5	NR	15; D	8
Goertz *et al*., 1990^27^	11	2 (18)	160	RT 11 (100)	11	1 (9)	8	7
Li *et al*., 2020^28^	50	0 (0)	(110–160)	CHT 26 (52)	4	NR	12	9
Li *et al*., 2016^30^	137	NR	NR	CHT 137 (100.0)	21	0 (0.0)	9.4; T	9
Montemaggi *et al*., 1991^44^	7	0 (0)	82 (60–100)	RT 4 (57)	NR	3 (43)	7; T	5
Morrow *et al*., 1984^34^	33	9 (27)	NR	NR	NR	8 (24)	8; T	4
Peretz *et al*., 1989^35^	98	0 (0)	136	CHT 27 (28), RT 27 (28)	9	NR	7	4
Schuricht *et al*., 1991^36^	42	0 (0)	(120–150)	CRT 42 (100)	24	NR	12.8	8
Shipley *et al*., 1980^46^	12	6 (50)	160	RT 12 (100)	5	NR	11	6
Syed *et al*., 1983^48^	18	1 (6)	(100–150)	RT 18 (100)	NR	4 (22)	14	7
Wang *et al*., 2013^50^	28	0 (0)	120	CHT 10 (36), RT 7 (25)	NR	NR	10.1	4
Wang *et al*., 2011^51^	27	15 (56)	(110–160)	CRT 6 (22), RT 1 (4)	NR	NR	8	4
Whittington *et al*., 1984^38^	33	0 (0)	120	CRT 20 (61), RT 13 (39)	37	NR	9; D	8
Zheng *et al*., 2017^54^	34	0 (0)	NR	CHT 8 (24)	6	NR	11; T	7
Total intraoperative	542	70 of 405 (17.3)	NA	CHT 208 (40.8), CRT 74 (14.5), RT 93 (18.3)	122 of 429	16 of 206 (7.8)	NA	NA
**Percutaneous**								
Chen *et al*., 2021^25^	22	4 (18)	130	CHT 22 (100)	22	NR	11.7; T	9
Chi *et al*., 2021^26^	21	0 (0)	130	CHT 21 (100)	24	0 (0)	13.2; T	4
Joyce *et al*., 1990^43^	19	NR	160	RT 12 (63)	22	NR	8.1; T	8
Liu *et al*., 2014^31^	30	0 (0)	NR	NR	6	NR	16	8
Lun *et al*., 2015^56^	38	NR	NR	CHT 38 (100)	NR	NR	11.8	4†
Luo *et al*., 2019^32^	35	NR	NR	CHT 35 (100)	NR	NR	9.5	7
Niu *et al*., 2016^45^	60	0 (0)	115 (110–130)	NR	NR	0 (0)	10.4	6
Wang *et al*., 2021^49^	28	NR	NR	NR	2	0 (0)	11.6	5
Wang *et al*., 2017^52^	32	25 (78)	120	CHT 16 (50)	NR	0 (0)	14	6
Yang *et al*., 2016^53^	18	0 (0)	167 (164–170)	CHT 18 (100), RT 1 (6)	10	3 (17)	7.3; T	5
Zhongmin *et al*., 2010^55^	31	12 (39)	52	CHT 10 (32)	NR	NR	10.3; T	7
Zhou *et al*., 2021^39^	67	0 (0)	NR	CHT 6 (9)	20	0 (0)	11; T	8
Total percutaneous	401	41 of 281 (14.6)	NA	CHT 166 (58.7), RT 13 (4.6)	106 of 205	3 of 226 (1.3)	NA	NA
**EUS**								
Du *et al*., 2013^41^	100	40 (40.0)	140 (120–210)	CHT 100 (100.0)	52	0 (0.0)	7; T	8
Jin *et al*., 2008^42^	22	8 (36)	NR	CHT 22 (100)	13	NR	9	6
Sun *et al*., 2006^47^	15	7 (47)	140	RT 1 (7)	31	4 (27)	10.6; T	8
Sun *et al*., 2017^37^	42	24 (57)	95	CHT 42 (100)	NR	0 (0)	9; T	2
Total EUS	179	79 of 179 (44.1)	NA	CHT 164 (91.6), RT 1 (0.6)	96 of 137	4 of 157 (2.5)	NA	NA
**Other**								
Li *et al*., 2020^29^	50	22 (44)	NR	CRT 6 (12)	17	0 (0)	8.8	8
Mohiuddin *et al*., 1994^33^	111	0 (0.0)	NR	CRT 81 (73.0), RT 30 (27.0)	NR	NR	11.4	3
Total (intraoperative, percutaneous, EUS, and other)	1283	212 of 1026 (20.7)	NA	CHT 538 (47.5), CRT 161 (14.2), RT 137 (12.1)	324 of 771	37 out of 600 (6.2)	NA	NA

Values are *n* (%) unless otherwise stated. *If the median was unavailable the mean is presented. †RCT. OS, overall survival; NOS, Newcastle–Ottawa scale; CRT, chemoradiotherapy; NR, not reported; D, diagnosis; RT, radiotherapy; CHT, chemotherapy; T, treatment; NA, not applicable; EUS, endoscopic ultrasonography.

In 27 studies reporting metastases, 212 of 1026 patients (20.7 per cent) had or developed stage IV pancreatic cancer. The included studies utilized ^125^I seeds with a length of 4.4 to 4.6 mm with a diameter of less than 1 mm^42,57^. Each patient received between 10 and 150 seeds in one or multiple operations depending on tumour volume, characteristics, and response. The median tumour dose ranged from 52 Gy^55^ to 167 Gy^53^, with most of the studies ranging between 100 and 150 Gy. For the application method, 14 studies (542 patients) implanted the seeds intraoperatively in an open approach using X-ray, CT, or ultrasonography guidance^27,28,30,34–36,38,40,44,46,48,50,51,54^. Twelve studies (401 patients) used percutaneous implantation guided by ultrasonography or CT^25,26,31,32,39,43,45,49,52,53,55,56^. Since 2006, four studies (179 patients) implemented endoscopic ultrasonography (EUS) to deliver the radioactive seeds to the tumour with a transgastric or transduodenal injection^37,41,42,47^. In 28 studies with 1132 patients, 538 patients (47.5 per cent), 161 patients (14.2 per cent), and 137 patients (12.1 per cent) received chemotherapy, chemoradiotherapy, or radiotherapy respectively.

Out of 600 patients in 15 studies reporting complications, 37 (6.2 per cent) suffered from greater than or equal to grade III complications (using Clavien–Dindo classification). Three studies reported postprocedural mortality^34,38,44^. Four deaths were caused by abscesses or anastomotic leakage^38^, three were caused by a pulmonary embolism^38,44^, two were caused by duodenal ulcers^38^, and one cause was not reported^34^. The most common complications reported were gastro­intestinal haemorrhages^35,38,46,54^, pancreatic fistula^34,35,44,54^, leuco­cytopenia^47^, and different intra-abdominal infections like pancrea­titis and cholangitis^35,38,42,47,48,54^. The median OS ranged from 7.0 months^35,41^ to 16 months^31^. In the RCT, no significant difference (*P* > 0.05) was found in adverse events between ^125^I combined with chemotherapy *versus* chemotherapy alone^56^. However, a statistically significant difference (*P <* 0.05*)* was established between the OS of ^125^I combined with chemotherapy (11.84 months) *versus* chemotherapy alone (10.40 months)^56^.

### Phosphorus-32 microbrachytherapy

An overview of the results of intratumoral injection ^32^P microbrachytherapy is presented in *Table 3*. All five included studies (133 patients) using ^32^P had a prospective design. One study compared ^32^P combined with previous 5-fluorouracil and gemcitabine chemotherapy after injection *versus* chemotherapy alone in an RCT^58^.

**Table 3:** Results of intratumoral phosphorus-32 microbrachytherapy for locally advanced pancreatic cancer

Reference	*n*	Metastasis	Type of ^32^P therapy	Tumour dose (Gy), median* (range)	Combination therapy	Overall complications, *n*	Complications ≥grade III	Median* survival (months); OS initiation	NOS 1–9
**Percutaneous**									
Order *et al*., 1996^59^	47	19 (40)	MAA and colloidal chromic ^32^P	(9000–17 000)	CRT 47 (100)	27	10 (53)	9.9; T	8
Rosemurgy *et al*., 2008^58^	18	0 (0)	Colloidal chromic ^32^P	1255	CRT 18 (100)	NR	16 (89)	5.2	8†
Westlin *et al*., 1997^61^	17	0 (0)	MAA and colloidal chromic ^32^P	5000 (1390–19 000)	CHT 2 (12)	NR	1 (6)	7.6; T	6
Total percutaneous	82	19 of 82 (23)	MAA and/or colloidal chromic 32P	NA	CHT 2 (2), CRT 65 (79)	27 of 47	27 of 82 (33)	NA	NA
**EUS**									
Bhutani *et al*., 2019^60^	9	0 (0)	Microparticle	100	CHT 9 (100)	NR	24 (NR)‡	NR	4
Ross *et al*., 2021^24^	42	0 (0)	Microparticle	100	CHT 42 (100)	1095	34 (81)	15.5; Inc	5
Total EUS	51	0 of 51 (0)	Microparticle	NA	CHT 51 (100)	1095 of 42	34 of 42 (81)	NA	NA
**Total**	133	19 of 133 (14.3)	MAA and/or colloidal chromic 32P or Microparticle	NA	CHT 53 (39.8), CRT 65 (48.9)	1122 of 89	61 of 124 (49.2)	NA	NA

Values are *n* (%) unless otherwise stated. *If the median was unavailable the mean is presented. †RCT. ‡*n* = number of complications. OS, overall survival; NOS, Newcastle–Ottawa scale; MAA, macroaggregated albumin; CRT, chemoradiotherapy; T, treatment; NR, not reported; CHT, chemotherapy; NA, not applicable; EUS, endoscopic ultrasonography; Inc, inclusion.

One study included 19 patients (40 per cent) with stage IV pancreatic cancer^59^. The remaining studies had no patients with metastases (total 14.3 per cent)^24,58,60,61^. ^32^P was only injected percutaneously with CT guidance and achieved a tumour dose between 1255 and 19 000 Gy^58,59,61^. This dose was a notably higher dose than the dose of 100 Gy achieved by the more recent microparticle brachy­therapy utilizing EUS application^24,60^.

Four studies (124 patients) reported complications. Some 61 patients (49.2 per cent) suffered from greater than or equal to grade III complications. The most frequently reported complications included haematological toxicities^24,58–60^, gastrointestinal haemorrhage^58,61^, fatigue^24,58^, and nausea^24,58^. The median OS of all five studies ranged from 5.2 months^58^ to 15.5 months after inclusion^24^. The RCT by Rosemurgy *et al*.^58^ (2008) was abandoned at a preliminary stage after treating 18 of 40 intended patients with ^32^P, due to a statistically significant higher complication rate (*P* = 0.03) and lower survival (*P* = 0.18) in the ^32^P group. The authors also found the highest complication rate, with 75 greater than or equal to grade III complications in 16 of 18 patients (89 per cent)^58^, followed closely by Ross *et al*.^24^ (2021) with 139 greater than or equal to grade III complications in 34 of 42 patients (81 per cent). In contrast, Ross *et al*.^24^ (2021) did find the highest survival of 15.5 months in the intratumoral injection ^32^P microbrachytherapy treatment group.

### Palladium-103 seed brachytherapy

An overview of the results of ^103^Pd seed brachytherapy is presented in *Table 4*. In 1996, two prospective studies applied ^103^Pd seed brachytherapy in 26 patients^62,63^.

**Table 4:** Results of intratumoral palladium-103 seed brachytherapy for locally advanced pancreatic cancer

Reference	*n*	Metastasis	Median* tumour dose (Gy)	Combination therapy	Overall complications, *n*	Complications ≥grade III	Median* survival (months); OS initiation	NOS 1–9
Nori *et al*., 1996^62^	15	0 (0)	110	CRT 15 (100)	NR	0 (0)	10; T	5
Raben *et al*., 1996^63^	11	1 (9)	124.4	CHT 2 (18), CRT 5 (45)	6	4 (36)	6.9	7
Total	26	1 of 26 (4)	NA	CHT 2 (8), CRT 20 (77)	6 of 26	4 of 26 (15)	NA	NA

Values are *n* (%) unless otherwise stated. *If the median was unavailable the mean is presented. OS, overall survival; NOS, Newcastle–Ottawa scale; CRT, chemoradiotherapy; NR, not reported; T, treatment; CHT, chemotherapy; NA, not applicable.

From the included 26 patients, one patient suffered stage IV pancreatic cancer^63^. On average, all patients were submitted to a dose of 110–124.2 Gy after intraoperative implantation. Two patients also underwent complementary chemotherapy^63^ and 20 underwent chemoradiotherapy^62,63^. Four patients suffered greater than or equal to grade III complications, including duodenal perforation, sepsis, cerebral vascular accident, and radiation enteritis^63^. An OS was found of 6.9 months^63^ and 10 months^62^.

### Immunotherapy

An overview of the results of intratumoral injection immunotherapy is presented in *Table 5*. Nine studies applied immunotherapy to 330 patients within a prospective design^64–72^, of which two were RCTs^67,71^. The two RCTs compared chemotherapy alone *versus* chemotherapy with an oncolytic virus^67,71^. The first RCT used TNFerade adenovirus with 5-fluorouracil chemoradiotherapy^67^ and the other one an H101 adenovirus for p53 activation with gemcitabine^71^.

**Table 5:** Results of intratumoral immunotherapy for locally advanced pancreatic cancer

Reference	*n*	Metastasis	Type of immunotherapy	Imaging	Combination therapy	Overall complications, *n*	Complications ≥grade III	Median* survival (months); OS initiation	NOS 1–9
**p53 activation pathway**									
Gong *et al*., 2011^64^	9	NR	H101 adenovirus	EUS (100)	CHT 9 (100)	NR	NR	7	7
Xiao *et al*., 2011^71^	19	NR	H101 adenovirus	EUS (100)	CHT 19 (100)	NR	NR	9	6†
Yunwei *et al*., 2010^72^	8	NR	H101 adenovirus	EUS (100)	CHT 8 (100)	NR	NR	6	3
Hecht *et al*., 2003^65^	21	12 (57)	ONYX-015 adenovirus	EUS (100)	CHT 21 (100)	NR	21 (100)	7.5; T	4
Li *et al*., 2011^69^	15	8 (53)	p53 adenovirus	Percutaneous ultrasonography (100)	CRT 15 (100)	64	8 (53)	13.8	7
Total p53 activation pathway	72	20 of 36 (56)	NA	EUS (79), Percutaneous ultrasonography (21)	CHT 57 (79), CRT 15 (21)	64 of 15	29 of 36 (81)	NA	NA
**Tumour necrosis factor-α pathway**									
Hecht *et al*., 2012^66^	50	0 (0)	TNFerade biologic	EUS (54), percutaneous (46)	CRT 50 (100)	NR	65 (NR)‡	9.9; Inc	8
Herman *et al*., 2013^67^	187	132 (70.6)	TNFerade biologic	EUS (50.8), Percutaneous, ultrasonography/CT (49.2)	CRT 187 (100.0)	219	116 (NR)‡	10; R	8†
Total tumour necrosis factor-α pathway	237	132 of 237 (55.7)	NA	EUS (51.5), percutaneous ultrasonography/CT (48.5)	CRT 237 (100.0)	219 of 187	NR	NA	NA
**Other immunotherapy**									
Hirooka *et al*., 2018^68^	15	0 (0)	Zoledronate-pulsed dendritic cells	EUS	CHT 15 (100)	33	4 (27)	11.5	8
Nishimura *et al*., 2018^70^	6	5 (83)	STNM01 oligonucleotide	EUS	None	0	0 (0)	5.8	5
**Total**	330	147 of 294 (50.0)	NA	NA	CHT 72 (21.8), CRT 252 (76.4)	316 of 223	33 of 57 (57.9)	NA	NA

Values are *n* (%) unless otherwise stated. *If the median was unavailable the mean is presented. †RCT. ‡*n* = number of complications. OS, overall survival; NOS, Newcastle–Ottawa scale; NR, not reported; EUS, endoscopic ultrasonography; CHT, chemotherapy; T, treatment; CRT, chemoradiotherapy; NA, not applicable; Inc, Inclusion; R, randomization.

Six studies reported metastases and half of the patients (147) had metastatic disease^65–70^. Five studies injected adenoviruses to increase p53 activation^64,65,69,71,72^. Two studies implanted TNFerade biologic, which enables tumour-specific delivery of TNF-α by radiation-inducible gene transfer^66,67^. One study injected zoledronate-pulsed dendritic cells combined with intravenous adoptive activated T lymphocytes to induce a CD8+ response^68^. Another study injected a double-stranded RNA oligonucleotide, called STNM01, to suppress a specific tumour growth factor (CHST15)^70^. In six studies the injection was guided by EUS^64,65,68,70–72^, in one study the injection was percutaneous^69^, and two studies used both methods^66,67^. From the 330 patients receiving immunotherapy, 72 patients (21.8 per cent) also underwent chemotherapy^64,65,68,71,72^ and 252 (76.4 per cent) underwent chemoradiotherapy^66,67,69^.

Four studies reported complication rates; 9 out of 36 patients (81 per cent) suffered greater than or equal to grade III complications after p53 adenovirus therapy^65,69^, four of 15 patients (27 per cent) suffered greater than or equal to grade III complications after zoledronate-pulsed dendritic cell injection^68^, and zero of 6 patients (0 per cent) suffered greater than or equal to grade III complications after STNM01 injection^70^. One study reported two cases of postprocedural mortality. One was caused by progressive disease and one was caused by a splenic artery thrombosis within 30 days post-intervention^66^. The most frequently presented complications included leucocytopenia^65,67–69^, severe pain^66,67^, fever^64,65,67–69,71,72^, gastrointestinal bleeding^66,67^, and intra-abdominal infection^66,67^. The median OS ranged between 5.8 months^70^ and 13.8 months^69^. In the first RCT, no significant difference in greater than or equal to grade II complications (*P* = 0.08) or OS (*P* = 0.26) was found between the TNFerade adenovirus injection combined with 5-fluorouracil chemoradiotherapy (greater than or equal to grade II complications 75.9 per cent, OS 10 months) and the chemoradiotherapy alone (greater than or equal to grade II complications 65.6 per cent, OS 10 months)^67^. The RCT applying H101 adenovirus for p53 activation did not report complications; this RCT found a significant difference (*P* = 0.004) in OS between the H101 adenovirus injection combined with gemcitabine (9 months) *versus* gemcitabine alone (6 months)^71^.

### Intratumoral chemotherapy

An overview of the results of intratumoral injection chemotherapy is presented in *Table 6*. Four prospective studies performed intratumoral chemotherapy in 71 patients^73–76^. One RCT studied a chemotherapy capsule implant (5-fluorouracil) combined with systemic chemotherapy of gemcitabine *versus* systemic gemcitabine alone^74^.

**Table 6:** Results of intratumoral chemotherapy for locally advanced pancreatic cancer

Reference	*n*	Metastasis	Type of intratumoral CHT	Intratumoral CHT dose (mg), median* (range)	Combination therapy	Overall complications, *n*	Complications ≥grade III	Median* survival (months); OS initiation	NOS 1–9
Levy *et al*., 2011^73^	36	11 (30)	Gemcitabine	90 (28–280)	CRT 36 (100)	0	0 (0)	9.3; T	5
Li *et al*., 2016^74^	18	NR	5-Fluorouracil capsule	(800–1500)	CHT 18 (100)	NR	NR	10.3	7†
Mohamadnejad *et al*., 2015^75^	12	0 (0)	Gemcitabine	168 (80–200)	CHT (NR), CRT (NR)	NR	0 (0)	9	8
Yang *et al*., 2017^76^	5	4 (80)	Gemcitabine	(400–600)	NR	3	0 (0)	16.2	7
			Cisplatin	(11.25–22.5)					
Total	71	15 of 53 (28)	NA	NA	CHT 18 (33), CRT 36 (67)	3 of 41	0 of 53 (0)	NA	NA

Values are *n* (%) unless otherwise stated. *If the median was unavailable the mean is presented. †RCT. CHT, chemotherapy; OS, overall survival; NOS, Newcastle–Ottawa scale; CRT, chemoradiotherapy; T, treatment; NR, not reported; NA, not applicable.

Three studies reported patients with metastases (15 of 53 patients (28 per cent))^73,75,76^. Gemcitabine injection was guided by EUS^73,75^. One study analysed the intratumoral distribution of percutaneous injection by injecting 1–2 ml of radiopaque agent before injecting gemcitabine and cisplatin with fibrin glue^76^. Capsules incorporating 5-fluorouracil were implanted intraoperatively followed by fibrin gel to prevent pancreatic fistula^74^. Two studies, including 54 patients, reported 18 patients (33 per cent) with combined systemic chemotherapy and 36 (67 per cent) with chemoradiotherapy^73,74^.

Three studies reported no occurrence of greater than or equal to grade III complications^73,75,76^. OS ranged from 9 months^75^ to 16.2 months^76^. The RCT did not report complication rates and no significant difference (*P* = 0.07) was found in the survival between treatment with implanted 5-fluorouracil capsules combined with systemic gemcitabine (10.3 months) *versus* systemic gemcitabine alone (8.1 months)^74^.

## Discussion

This systematic review reveals data on five types of intratumoral injection therapy with widely heterogeneous safety and survival outcomes in patients with LAPC.

^125^I brachytherapy, intratumoral chemotherapy, and ^103^Pd brachytherapy are associated with low rates of greater than or equal to grade III complications in the current literature review. In contrast, the complication rates of ^32^P brachytherapy and intratumoral immunotherapy were at least three-fold higher. Within the ^32^P and immunotherapy intervention groups, less complications seemed to be related to the injection procedure and more to the injected agents^24,60,61,64,68,69^. A common procedure-related complication was bacterial infection from the gastrointestinal tract into the pancreas, which was easily treated with antibiotics^65^. For immunotherapy, the method of injection (EUS or percutaneous) did not seem to influence complication rates^65,66^. An explanation for the high severe complication rate after immunotherapy is the triggered autoimmune response. After immunotherapy, intra-abdominal infection^66,67^ and fever^64,65,67–69,71,72^ were often observed. These are clear signs of an autoimmune response^77^. Current research into upcoming immuno­therapies also attempts to identify and control these side effects^78^. The high complication rate after ^32^P microbrachytherapy is possibly due to a radiation overdose and therapy diffusion into healthy tissues^58^. Rosemurgy *et al*.^58^ (2008) reported therapy diffusion of ^32^P into nearby tissues and found a high complication rate (89 per cent), whereas Westlin *et al*.^61^ (1997) did not report therapy diffusion and found a much lower complication rate (6 per cent) with an exceptionally higher median tumour dose (1227 *versus* 11 050 Gy respectively). Ross *et al*.^24^ (2021) also found a high complication rate (81 per cent) after a median tumour dose of only 100 Gy (±20 per cent); however, they also claimed that only 8 of 139 (5.8 per cent) severe complications were ^32^P or procedure related, and reported that almost no therapy diffusion occurred outside the tumour. These heterogeneous results might suggest that, with therapy deposition central within the tumour, possibly with image guidance for improved treatment control and clear safety margins, microbrachytherapy could still prove to be a safe treatment method for LAPC.

The rate of severe complications after ^125^I brachytherapy, intratumoral chemotherapy, and ^103^Pd brachytherapy was below the complication rate of the most common ablative treatment for LAPC (radiofrequency ablation (RFA)). Rombouts *et al*.^16^ (2015) published a systematic review concerning ablative treatment methods for LAPC. In the RFA group an overall complication rate of 24.2 per cent was found, with a 13.6 per cent RFA-procedure-related complication rate^16^.

LAPC patients undergoing intratumoral injection therapy are generally also treated with systemic chemotherapy. Systemic chemotherapy is associated with side-effects, such as leucocytopenia and thrombo­cytopenia^24,47,58–60,65,67–69,79^. Even after modern chemotherapy regimens, such as FOLFIRINOX, complication rates range from 19.1–23.2 per cent^80^ up to 50 per cent^11^. Whether complications are related to intratumoral injection therapy or systemic chemotherapy can be difficult to identify. Overall, 11 cases of short-term postprocedural mortality were reported. Three RCTs compared complication rates of systemic chemotherapy combined with intratumoral injection therapy *versus* systemic chemotherapy alone. Two found no significant difference (^125^I and TNFerade)^56,67^ and one did find a significant difference with disadvantage towards ^32^P^58^. An RCT with modern chemotherapy regimens, with and without intratumoral injection therapy, should be the cornerstone to assess safety in this patient population.

The survival outcomes of the intratumoral injection modalities varied considerably between 5.0 and 16.2 months. No single intratumoral injection modality showed consistent high survival outcomes. Regarding survival outcome, Ross *et al*.^24^ (2021) showed the most promising results with a median OS of 15.5 months in 42 patients after receiving ^32^P microbrachytherapy. Considering the absence of a control group and, therefore, the high chance of selection bias, the benefit of ^32^P is still questionable^24^.

With regards to ablative treatment, Rombouts *et al*.^16^ (2015) found an OS of between 5.0 and 25.6 months in the RFA group. The highest survival of 25.6 months was found when RFA was combined with several different therapies, including intra-arterial plus systemic chemotherapy^81^. When RFA was applied as monotherapy, the median survival was 14.7 months. Still, evident selection bias was present^81^. More recent studies applying RFA for LAPC patients found a survival between 5.0 and 9.0 months with and without a combination of chemo­therapy^82,83^. Overall, similar survival results are shown for most intratumoral therapies in this review.

Whether intratumoral injection therapy contributes to the survival of patients with LAPC remains questionable with the currently available literature. Due to the insidious onset and probable microscopic spread at the time of diagnosis, pancreatic cancer is essentially a systemic disease and local therapies may not contribute to survival^36^. Even if no metastases are found at the time of diagnosis, the disease may have already spread to the pancreatic surroundings. The OS results of the current review substantiate this theory by showing slightly improved survival after chemotherapy compared with no chemotherapy in the ^125^I group and similar survival between studies with and without metastatic disease^69,76^. The potential clinical benefit of local tumour therapy in patients with pancreatic cancer is not limited to survival. Local tumour response and local progression-free survival can be of great value for patients, especially when providing pain relief and improving and prolonging the performance score and quality of life.

The included studies have several limitations. Most studies were case series and cohort studies with small sample sizes. Selection bias in several forms hampers the quality of the studies, such as the type of LAPC classification guideline (NCCN or AJCC)^17^, local diagnostic and treatment protocols, additional diagnostic research, and prior treatment completion. To take selection bias into consideration, the NOS was applied.

To present a clear overview, many results had to be filtered or adjusted to fit certain classifications. Therefore, undetailed data were often excluded from the analysis.

Combination therapies have been categorized by the type of therapy (for example chemotherapy, chemoradiotherapy, or radiotherapy) and not by the technical aspects, start, duration, dose, and iteration of the cycles. Even though all studies, except one, used gemcitabine-based chemotherapy, the current movement towards FOLFIRINOX-based chemotherapy might have a radical impact on oncological outcomes soon. Metastases were present at different rates, locations, and quantities within each study. Additionally, studies were not excluded if metastatic disease was present or occurred in a minority of the included patients. A large variation in survival was seen, which could partly be explained by the moment from which survival was measured (for example the initial diagnosis, inclusion in the study, or the intervention); however, this was not consistently reported. Potential differences in lead time of several months may have had a great impact on OS differences.

Five intratumoral injection therapies are described and an overview is reported. Some intratumoral injection therapies for patients with LAPC seem safe, although ^32^P microbrachytherapy and immunotherapy require additional evidence. Currently available data on all modalities are insufficient to provide firm conclusions regarding the added value to survival. Clinical benefits of these procedures are potentially not limited to survival, but control of local tumour growth could be of great value for patients, especially when providing pain relief and improving quality of life. The potential advantage of intratumoral injection therapies complementary to conventional care should therefore be studied in well designed RCTs.

## Supplementary Material

zrad052_Supplementary_Data

## Data Availability

Research data can be made available on request.

## References

[1] Lippi G , Mattiuzzi C. The global burden of pancreatic cancer. Arch Med Sci 2020;16:820–824 32542083 10.5114/aoms.2020.94845 PMC7286317

[2] Rawla P , Sunkara T, Gaduputi V. Epidemiology of pancreatic cancer: global trends, etiology and risk factors. World J Oncol 2019;10:10–27 30834048 10.14740/wjon1166 PMC6396775

[3] American Cancer Society . Cancer Facts and Figures 2020. cancer.org/content/dam/cancer-org/research/cancer-facts-and-statistics/annual-cancer-facts-and-figures/2020/cancer-facts-and-figures-2020.pdf (accessed 29 June 2020)

[4] Keane MG , Bramis K, Pereira SP, Fusai GK. Systematic review of novel ablative methods in locally advanced pancreatic cancer. World J Gastroenterol 2014;20:2267–2278 24605026 10.3748/wjg.v20.i9.2267 PMC3942832

[5] Siegel RL , Miller KD, Jemal A. Cancer statistics, 2018. CA Cancer J Clin 2018;68:7–30 29313949 10.3322/caac.21442

[6] Tempero MA , Malafa MP, Al-Hawary M, Asbun H, Bain A, Behrman SW et al Pancreatic Adenocarcinoma, Version 2.2017, NCCN Clinical Practice Guidelines in Oncology. J Natl Compr Canc Netw 2017;15:1028–1061 28784865 10.6004/jnccn.2017.0131

[7] Verslype C , Van Cutsem E, Dicato M, Cascinu S, Cunningham D, Diaz-Rubio E et al The management of pancreatic cancer. Current expert opinion and recommendations derived from the 8th World Congress on Gastrointestinal Cancer, Barcelona, 2006. Ann Oncol 2007;18(Suppl 7):vii1–vii10 17600091 10.1093/annonc/mdm210

[8] Dutch Federation of Medical Specialists (Federatie Medisch Specialisten) . Pancreas Carcinoma. richtlijnendatabase.nl/richtlijn/pancreascarcinoom/ (accessed 17 June 2020)

[9] Gemenetzis G , Groot VP, Blair AB, Laheru DA, Zheng L, Narang AK et al Survival in locally advanced pancreatic cancer after neoadjuvant therapy and surgical resection. Ann Surg 2019;270:340–347 29596120 10.1097/SLA.0000000000002753 PMC6985003

[10] Brada LJH , Walma MS, Daamen LA, van Roessel S, van Dam RM, de Hingh IH et al Predicting overall survival and resection in patients with locally advanced pancreatic cancer treated with FOLFIRINOX: development and internal validation of two nomograms. J Surg Oncol 2021;124:589–597 34115379 10.1002/jso.26567

[11] Rombouts SJ , Mungroop TH, Heilmann MN, van Laarhoven HW, Busch OR, Molenaar IQ et al FOLFIRINOX in locally advanced and metastatic pancreatic cancer: a single centre cohort study. J Cancer 2016;7:1861–1866 27698926 10.7150/jca.16279 PMC5039370

[12] Suker M , Beumer BR, Sadot E, Marthey L, Faris JE, Mellon EA et al FOLFIRINOX for locally advanced pancreatic cancer: a systematic review and patient-level meta-analysis. Lancet Oncol 2016;17:801–810 27160474 10.1016/S1470-2045(16)00172-8 PMC5527756

[13] Walma MS , Brada LJ, Patuleia SIS, Blomjous JG, Bollen TL, Bosscha K et al Treatment strategies and clinical outcomes in consecutive patients with locally advanced pancreatic cancer: a multicenter prospective cohort. Eur J Surg Oncol 2021;47:699–707 33280952 10.1016/j.ejso.2020.11.137

[14] Chen YG , Pan HH, Dai MS, Lin C, Lu CS, Su SL et al Impact of comorbidity and age on determinants therapeutic strategies in advanced pancreatic head cancer patients with obstructive jaundices. Medicine (Baltimore) 2015;94:e1298 26252308 10.1097/MD.0000000000001298 PMC4616572

[15] Parmar AD , Vargas GM, Tamirisa NP, Sheffield KM, Riall TS. Trajectory of care and use of multimodality therapy in older patients with pancreatic adenocarcinoma. Surgery 2014;156:280–289 24851723 10.1016/j.surg.2014.03.001 PMC4099282

[16] Rombouts SJ , Vogel JA, van Santvoort HC, van Lienden KP, van Hillegersberg R, Busch OR et al Systematic review of innovative ablative therapies for the treatment of locally advanced pancreatic cancer. Br J Surg 2015;102:182–193 25524417 10.1002/bjs.9716

[17] He J , Page AJ, Weiss M, Wolfgang CL, Herman JM, Pawlik TM. Management of borderline and locally advanced pancreatic cancer: where do we stand? World J Gastroenterol 2014;20:2255–2266 24605025 10.3748/wjg.v20.i9.2255 PMC3942831

[18] Jusoh AC , Ammori BJ. Laparoscopic versus open distal pancreatectomy: a systematic review of comparative studies. Surg Endosc 2012;26:904–913 22083328 10.1007/s00464-011-2016-3

[19] Liberati A , Altman DG, Tetzlaff J, Mulrow C, Gotzsche PC, Ioannidis JP et al The PRISMA statement for reporting systematic reviews and meta-analyses of studies that evaluate health care interventions: explanation and elaboration. PLoS Med 2009;6:e1000100 10.1371/journal.pmed.1000100 PMC2707010 19621070

[20] National Comprehensive Cancer Network (NCCN) . NCCN Clinical Practice Guidelines in Oncology (NCCN Guidelines^®^): Pancreatic Adenocarcinoma. Version 1.2022, 24 February 2022

[21] SCImago . SJR — SCImago Journal & Country Rank. scimagojr.com (accessed 23 March 2020)

[22] Dindo D , Demartines N, Clavien PA. Classification of surgical complications: a new proposal with evaluation in a cohort of 6336 patients and results of a survey. Ann Surg 2004;240:205–213 15273542 10.1097/01.sla.0000133083.54934.ae PMC1360123

[23] Eisenhauer EA , Therasse P, Bogaerts J, Schwartz LH, Sargent D, Ford R et al New response evaluation criteria in solid tumours: revised RECIST guideline (version 1.1). Eur J Cancer 2009;45:228–247 19097774 10.1016/j.ejca.2008.10.026

[24] Ross PJ , Wasan HS, Croagh D, Nikfarjam M, Nguyen N, Aghmesheh M et al Results of a single-arm pilot study of ^32^P microparticles in unresectable locally advanced pancreatic adenocarcinoma with gemcitabine/nab-paclitaxel or FOLFIRINOX chemotherapy. ESMO Open 2021;7:100356 10.1016/j.esmoop.2021.100356 PMC8717429 34953400

[25] Chen C , Wang W, Wang Y, Yu Z, Li Y. Locally advanced pancreatic carcinoma with jaundice: the benefit of a sequential treatment with stenting followed by CT-guided ^125^I seeds implantation. Eur Radiol 2021;31:6500–6510 33630162 10.1007/s00330-021-07764-6 PMC8379103

[26] Chi Z , Chen L, Huang J, Jiang N, Zheng Q, Huang N et al A novel combination of percutaneous stenting with iodine-125 seed implantation and chemotherapy for the treatment of pancreatic head cancer with obstructive jaundice. Brachytherapy 2021;20:218–225 33158777 10.1016/j.brachy.2020.09.009

[27] Goertz SR , Ali MM, Parker GA. Local management of pancreatic carcinoma: iodine-125 implantation. Clin Oncol (R Coll Radiol) 1990;2:22–26 1702011 10.1016/s0936-6555(05)80214-6

[28] Li CG , Zhou ZP, Jia YZ, Tan XL, Song YY. Radioactive ^125^I seed implantation for locally advanced pancreatic cancer: a retrospective analysis of 50 cases. World J Clin Cases 2020;8:3743–3750 32953850 10.12998/wjcc.v8.i17.3743 PMC7479562

[29] Li W , Wang X, Wang Z, Zhang T, Cai F, Tang P et al The role of seed implantation in patients with unresectable pancreatic carcinoma after relief of obstructive jaundice using ERCP. Brachytherapy 2020;19:97–103 31564517 10.1016/j.brachy.2019.08.010

[30] Li YF , Liu ZQ, Zhang YS, Dong LM, Wang CY, Gou SM et al Implantation of radioactive ^125^I seeds improves the prognosis of locally advanced pancreatic cancer patients: a retrospective study. J Huazhong Univ Sci Technolog Med Sci 2016;36:205–210 27072963 10.1007/s11596-016-1567-x

[31] Liu K , Ji B, Zhang W, Liu S, Wang Y, Liu Y. Comparison of iodine-125 seed implantation and pancreaticoduodenectomy in the treatment of pancreatic cancer. Int J Med Sci 2014;11:893–896 25013369 10.7150/ijms.8948 PMC4081311

[32] Luo M , Zhang F. CT-guided ^125^I brachytherapy combined with transarterial infusion for the treatment of unresectable or locally advanced pancreatic carcinoma. Brachytherapy 2019;18(Suppl):S83–SS4 10.5152/dir.2020.19371 PMC7837730 33252336

[33] Mohiuddin M , Rosato F, Schuricht A, Barbot D, Biermann W, Cantor R. Carcinoma of the pancreas–the Jefferson experience 1975-1988. Eur J Surg Oncol 1994;20:13–20 8131862

[34] Morrow M , Hilaris B, Brennan MF. Comparison of conventional surgical resection, radioactive implantation, and bypass procedures for exocrine carcinoma of the pancreas 1975–1980. Ann Surg 1984;199:1–5 6419687 10.1097/00000658-198401000-00001 PMC1353249

[35] Peretz T , Nori D, Hilaris B, Manolatos S, Linares L, Harrison L et al Treatment of primary unresectable carcinoma of the pancreas with I-125 implantation. Int J Radiat Oncol Biol Phys 1989;17:931–935 2808054 10.1016/0360-3016(89)90138-7

[36] Schuricht AL , Barbot DJ, Mohiuddin M, Rosato FE. Adenocarcinoma of the pancreas: a multimodality approach–a single surgeon's experience (1979–1988). J Surg Oncol 1991;48:56–61 1716332 10.1002/jso.2930480111

[37] Sun X , Lu Z, Wu Y, Min M, Bi Y, Shen W et al An endoscopic ultrasonography-guided interstitial brachytherapy based special treatment-planning system for unresectable pancreatic cancer. Oncotarget 2017;8:79099–79110 29108290 10.18632/oncotarget.15763 PMC5668023

[38] Whittington R , Solin L, Mohiuddin M, Cantor RI, Rosato FE, Biermann WA et al Multimodality therapy of localized unresectable pancreatic adenocarcinoma. Cancer 1984;54:1991–1998 6478433 10.1002/1097-0142(19841101)54:9<1991::aid-cncr2820540934>3.0.co;2-4

[39] Zhou S , Zhu C, Chen SL, Li JA, Qu KL, Jing H et al ^125^I intracavitary irradiation combined with ^125^I seeds implantation for treatment of locally advanced pancreatic head cancer: a retrospective analysis of 67 cases. Int J Gen Med 2021;14:2645–2653 34177273 10.2147/IJGM.S309069 PMC8219295

[40] Dobelbower RR Jr , Merrick HW 3rd, Ahuja RK, Skeel RT. ^125^I interstitial implant, precision high-dose external beam therapy, and 5-FU for unresectable adenocarcinoma of pancreas and extrahepatic biliary tree. Cancer 1986;58:2185–2195 3756765 10.1002/1097-0142(19861115)58:10<2185::aid-cncr2820581004>3.0.co;2-q

[41] Du Y , Jin Z, Meng H, Zou D, Chen J, Liu Y et al Long-term effect of gemcitabine-combined endoscopic ultrasonography-guided brachytherapy in pancreatic cancer. J Interv Gastroenterol 2013;3:18–24

[42] Jin Z , Du Y, Li Z, Jiang Y, Chen J, Liu Y. Endoscopic ultrasonography-guided interstitial implantation of iodine 125-seeds combined with chemotherapy in the treatment of unresectable pancreatic carcinoma: a prospective pilot study. Endoscopy 2008;40:314–320 18283622 10.1055/s-2007-995476

[43] Joyce F , Burcharth F, Holm HH, Stroyer I. Ultrasonically guided percutaneous implantation of iodine-125 seeds in pancreatic carcinoma. Int J Radiat Oncol Biol Phys 1990;19:1049–1052 1698755 10.1016/0360-3016(90)90032-f

[44] Montemaggi P , Dobelbower R, Crucitti F, Caracciolo F, Morganti AG, Smaniotto D et al Interstitial brachytherapy for pancreatic cancer: report of seven cases treated with ^125^I and a review of the literature. Int J Radiat Oncol Biol Phys 1991;21:451–457 2061121 10.1016/0360-3016(91)90795-6

[45] Niu L . Combination of iodine-125 seed implantation with cryosurgery for locally advanced pancreatic carcinoma. Brachytherapy 2016;15(Suppl 1):S141

[46] Shipley WU , Nardi GL, Cohen AM, Ling CC. Iodine-125 implant and external beam irradiation in patients with localized pancreatic carcinoma: a comparative study to surgical resection. Cancer 1980;45:709–714 6244074 10.1002/1097-0142(19800215)45:4<709::aid-cncr2820450416>3.0.co;2-5

[47] Sun S , Xu H, Xin J, Liu J, Guo Q, Li S. Endoscopic ultrasound-guided interstitial brachytherapy of unresectable pancreatic cancer: results of a pilot trial. Endoscopy 2006;38:399–403 16680642 10.1055/s-2006-925253

[48] Syed AM , Puthawala AA, Neblett DL. Interstitial iodine-125 implant in the management of unresectable pancreatic carcinoma. Cancer 1983;52:808–813 6191854 10.1002/1097-0142(19830901)52:5<808::aid-cncr2820520510>3.0.co;2-u

[49] Wang B . Individualized 3D-printing coplanar template-assisted iodine-125 seed implantation for inoperable pancreatic cancer. Brachytherapy 2021;20(Suppl):S83 10.5114/jcb.2022.114990 PMC9044303 35494180

[50] Wang H , Junjie W, Yuliang J, Jinna L, Suqing T, Weiqiang R et al Prognostic factors for intraoperative ^125^I seeds implantation for treatment of locally-advanced pancreatic carcinoma. Int J Radiat Oncol Biol Phys 2013;87(Suppl):S310

[51] Wang J , Li J, Tian S, Jiang Y, Ran W, Xiu D. Intraoperative ultrasound-guided ^125^I implantation in the treatment of unresectable pancreatic carcinoma. Brachytherapy 2011;10(Suppl):S70

[52] Wang W , Wang Y, Li Y. CT-guided iodine-125 seeds implantation combined with chemotherapy for locally advanced pancreatic carcinoma. Brachytherapy 2017;16(Suppl):S47

[53] Yang M , Yan Z, Luo J, Liu Q, Zhang W, Ma J et al A pilot study of intraluminal brachytherapy using ^125^I seed strand for locally advanced pancreatic ductal adenocarcinoma with obstructive jaundice. Brachytherapy 2016;15:859–864 27364870 10.1016/j.brachy.2016.05.004

[54] Zheng Z , Xu Y, Zhang S, Pu G, Cui C. Surgical bypass and permanent iodine-125 seed implantation vs. surgical bypass for the treatment of pancreatic head cancer. Oncol Lett 2017;14:2838–2844 28927042 10.3892/ol.2017.6495 PMC5588109

[55] Zhongmin W , Yu L, Fenju L, Kemin C, Gang H. Clinical efficacy of CT-guided iodine-125 seed implantation therapy in patients with advanced pancreatic cancer. Eur Radiol 2010;20:1786–1791 20069424 10.1007/s00330-009-1703-0

[56] Lun J-J , Zhao J-L, Sun J-Y, Hu X-K, Yin H-Z. CT-guided ^125^I radioactive seed interstitial implantation combined with chemotherapy for advanced pancreatic carcinoma: analysis of therapeutic efficacy. J Interventional Radiol (China) 2015;24:494–497

[57] Heintz BH , Wallace RE, Hevezi JM. Comparison of I-125 sources used for permanent interstitial implants. Med Phys 2001;28:671–682 11339765 10.1118/1.1359246

[58] Rosemurgy A , Luzardo G, Cooper J, Bowers C, Zervos E, Bloomston M et al ^32^P as an adjunct to standard therapy for locally advanced unresectable pancreatic cancer: a randomized trial. J Gastrointest Surg 2008;12:682–688 18266048 10.1007/s11605-007-0430-6

[59] Order SE , Siegel JA, Principato R, Zeiger LE, Johnson E, Lang P et al Selective tumor irradiation by infusional brachytherapy in nonresectable pancreatic cancer: a phase I study. Int J Radiat Oncol Biol Phys 1996;36:1117–1126 8985034 10.1016/s0360-3016(96)00484-1

[60] Bhutani MS , Klapman JB, Tuli R, El-Haddad GE, Hoffe S, Wong FCL et al OncoPaC-1: an open-label, single-arm pilot study of phosphorus-32 microparticles brachytherapy in combination with gemcitabine +/- nab-paclitaxel in unresectable locally advanced pancreatic cancer. Int J Radiat Oncol Biol Phys 2019;105(Suppl):E236–E237 10.4103/eus.eus_44_19 PMC7038730 31670288

[61] Westlin JE , Andersson-Forsman C, Garske U, Linne T, Aas M, Glimelius B et al Objective responses after fractionated infusional brachytherapy of unresectable pancreatic adenocarcinomas. Cancer 1997;80:2743–2748 9406733 10.1002/(sici)1097-0142(19971215)80:12+<2743::aid-cncr54>3.3.co;2-4

[62] Nori D , Merimsky O, Osian AD, Heffernan M, Cortes E, Turner JW. Palladium-103: a new radioactive source in the treatment of unresectable carcinoma of the pancreas: a phase I-II study. J Surg Oncol 1996;61:300–305 8628003 10.1002/(SICI)1096-9098(199604)61:4<300::AID-JSO14>3.0.CO;2-9

[63] Raben A , Mychalczak B, Brennan MF, Minsky B, Anderson L, Casper ES et al Feasibility study of the treatment of primary unresectable carcinoma of the pancreas with ^103^Pd brachytherapy. Int J Radiat Oncol Biol Phys 1996;35:351–356 8635943 10.1016/0360-3016(95)02136-1

[64] Gong T , Zhu Q, Zhang Y, Xu K, Chen X, Wu J et al Study on EUS guided oncolytic adenovirus implantation in patients with unresectable pancreatic cancer. Digestion 2011;83:235

[65] Hecht JR , Bedford R, Abbruzzese JL, Lahoti S, Reid TR, Soetikno RM et al A phase I/II trial of intratumoral endoscopic ultrasound injection of ONYX-015 with intravenous gemcitabine in unresectable pancreatic carcinoma. Clin Cancer Res 2003;9:555–561 12576418

[66] Hecht JR , Farrell JJ, Senzer N, Nemunaitis J, Rosemurgy A, Chung T et al EUS or percutaneously guided intratumoral TNFerade biologic with 5-fluorouracil and radiotherapy for first-line treatment of locally advanced pancreatic cancer: a phase I/II study. Gastrointest Endosc 2012;75:332–338 22248601 10.1016/j.gie.2011.10.007 PMC4380192

[67] Herman JM , Wild AT, Wang H, Tran PT, Chang KJ, Taylor GE et al Randomized phase III multi-institutional study of TNFerade biologic with fluorouracil and radiotherapy for locally advanced pancreatic cancer: final results. J Clin Oncol 2013;31:886–894 23341531 10.1200/JCO.2012.44.7516 PMC4820756

[68] Hirooka Y , Kawashima H, Ohno E, Ishikawa T, Kamigaki T, Goto S et al Comprehensive immunotherapy combined with intratumoral injection of zoledronate-pulsed dendritic cells, intravenous adoptive activated T lymphocyte and gemcitabine in unresectable locally advanced pancreatic carcinoma: a phase I/II trial. Oncotarget 2018;9:2838–2847 29416816 10.18632/oncotarget.22974 PMC5788684

[69] Li JL , Cai Y, Zhang SW, Xiao SW, Li XF, Duan YJ et al Combination of recombinant adenovirus-p53 with radiochemotherapy in unresectable pancreatic carcinoma. Chin J Cancer Res 2011;23:194–200 23467436 10.1007/s11670-011-0194-0 PMC3587558

[70] Nishimura M , Matsukawa M, Fujii Y, Matsuda Y, Arai T, Ochiai Y et al Effects of EUS-guided intratumoral injection of oligonucleotide STNM01 on tumor growth, histology, and overall survival in patients with unresectable pancreatic cancer. Gastrointest Endosc 2018;87:1126–1131 29122598 10.1016/j.gie.2017.10.030

[71] Xiao B , Jin ZD, Du YQ, Wu RP, Li ZS. Intratumoral injection of E1B gene-deleted adenovirus combined with intravenous gemcitabine in treating unresectable pancreatic carcinoma. J Gastroenterol Hepatol 2011;26:12–13

[72] Yunwei S , Qi Z, Kai X, Xi C, Lu X, Ji-Hong T. Preliminary studies of EUS guided oncolytic adenovirus implantation combined with chemotherapy in patients of non-operative pancreatic cancer. Digestion 2010;81:166

[73] Levy MJ , Alberts SR, Chari ST, Farnell MB, Haddock MG, Kendrick ML et al EUS guided intra-tumoral gemcitabine therapy for locally advanced and metastatic pancreatic cancer. Gastrointest Endosc 2011;73(Suppl):AB144–AB145 10.1016/j.gie.2016.11.014 PMC6131689 27889543

[74] Li JQ , Yang JC, Liang JX, Wang SL. Pharmacokinetic study and clinical evaluation of a slow-release 5-fluorouracil implant in pancreatic cancer patients. Anticancer Drugs 2016;27:60–65 26375684 10.1097/CAD.0000000000000293

[75] Mohamadnejad M , Zamani F, Setareh M, Nikfam S, Malekzadeh R. EUS-guided intratumoral gemcitabine injection in locally advanced non-metastatic pancreatic cancer. Gastrointest Endosc 2015;81(Suppl):AB440–AB441

[76] Yang B , He JP, Yuan ML, Li W, Jiao H, You X et al Percutaneous intratumoral injection of gemcitabine plus cisplatin mixed with fibrin glue for advanced pancreatic carcinoma: case report. Medicine (Baltimore) 2017;96:e8018 28906385 10.1097/MD.0000000000008018 PMC5604654

[77] Barber FD . Adverse events of oncologic immunotherapy and their management. Asia Pac J Oncol Nurs 2019;6:212–226 31259216 10.4103/apjon.apjon_6_19 PMC6518984

[78] National Cancer Institute . New Drugs, New Side Effects: Complications of Cancer Immunotherapy. cancer.gov/news-events/cancer-currents-blog/2019/cancer-immunotherapy-investigating-side-effects (accessed 11 August 2020)

[79] Moysan E , Bastiat G, Benoit JP. Gemcitabine versus modified gemcitabine: a review of several promising chemical modifications. Mol Pharm 2013;10:430–444 22978251 10.1021/mp300370t

[80] Rombouts SJ , Walma MS, Vogel JA, van Rijssen LB, Wilmink JW, Mohammad NH et al Systematic review of resection rates and clinical outcomes after FOLFIRINOX-based treatment in patients with locally advanced pancreatic cancer. Ann Surg Oncol 2016;23:4352–4360 27370653 10.1245/s10434-016-5373-2 PMC5090009

[81] Cantore M , Girelli R, Mambrini A, Frigerio I, Boz G, Salvia R et al Combined modality treatment for patients with locally advanced pancreatic adenocarcinoma. Br J Surg 2012;99:1083–1088 22648697 10.1002/bjs.8789

[82] Fegrachi S , Walma MS, de Vries JJJ, van Santvoort HC, Besselink MG, von Asmuth EG et al Safety of radiofrequency ablation in patients with locally advanced, unresectable pancreatic cancer: a phase II study. Eur J Surg Oncol 2019;45:2166–2172 31227340 10.1016/j.ejso.2019.06.008

[83] He C , Wang J, Zhang Y, Cai Z, Lin X, Li S. Comparison of combination therapies in the management of locally advanced pancreatic cancer: induction chemotherapy followed by irreversible electroporation vs radiofrequency ablation. Cancer Med 2020;9:4699–4710 32410380 10.1002/cam4.3119 PMC7333834

