# Supplementary material for: Intratumoral injection therapies for locally advanced pancreatic cancer: systematic review
Source: BJS Open. 2023 May 31;7(3):zrad052. doi: 10.1093/bjsopen/zrad052 (PMC10230443; doi:10.1093/bjsopen/zrad052)
Supplement: zrad052_Supplementary_Data [file zrad052_supplementary_data.zip › Supplementary_Material.docx]

**Intratumoral injection therapies for locally advanced pancreatic cancer: systematic review**

Coen Ysbrand Willink^1*^, Sjoerd Franciscus Maria Jenniskens^1^, Nienke Johanna Maria Klaassen^1^**,** Martijn Willem Jan Stommel^2^, Johannes Frank Wilhelmus Nijsen^1^

^1^Department of Medical Imaging, Radboud Institute for Health Sciences, Radboud University Medical Centre, Nijmegen, The Netherlands

^2^Department of Surgery, Radboud Institute for Health Sciences, Radboud University Medical Centre, Nijmegen, The Netherlands.

*Correspondence: Department of Medical imaging, M330.-1.003, Radboud University Medical Centre, Geert Grooteplein Zuid 10, 6525 GA Nijmegen, The Netherlands (e-mail: [Ysbrand.Willink@radboudumc.nl](mailto:Ysbrand.Willink@radboudumc.nl)

**Supplementary Materials - Index**

| **Supplementary Appendixes** |  |
| --- | --- |
| Appendix S1 – Search Strategy | *pag. 2-4* |
| Appendix S2 – Newcastle Ottawa Quality Assessment Scale  Appendix S3 – Newcastle Ottawa Quality Assessment Scale Results | *pag. 5-6*  *pag. 7-8* |
| Appendix S4 – An overview of applied radioactive isotopes for intratumoral injection therapies in pancreatic cancer | *Pag. 9* |

# Supplementary Appendixes

# Appendix S1 – Search strategy

**PubMed**

"Pancreatic Neoplasms"[Mesh] OR

Pancreatic Neoplasm*[tiab] OR

Pancreatic adeno*[tiab] OR

Pancreas adeno*[tiab] OR

Pancreas Neoplasm*[tiab] OR

Pancreatic carcino*[tiab] OR

Pancreas carcino*[tiab] OR

Pancreas tum*[tiab] OR

Pancreatic tum*[tiab] OR

Cancer of Pancreas[tiab] OR

Tumour of the pancreas[tiab] OR

Pancreas Cancer*[tiab] OR

Pancreatic Cancer*[tiab] OR

Cancer of the Pancreas[tiab] OR

Carcinoma of the pancreas[tiab] OR

Islet Cell Adenoma* [tiab] OR

Insulinoma*[tiab] OR

Islet Cell Carcinoma* [tiab] OR

Gastrinoma*[tiab] OR

Glucagonoma*[tiab] OR

Somatostatinoma*[tiab] OR

Vipoma*[tiab] OR

Pancreatic Ductal Carcino* [tiab] OR

Pancreatic Ductal Adeno*[tiab] OR

Pancreatic Ductal Neoplasm*[tiab] OR

Pancreatic Ductal Cancer*[tiab] OR

Pancreatic Intraductal Carcino*[tiab] OR

Pancreatic Intraductal Adeno*[tiab] OR

Pancreatic Intraductal Neoplasm*[tiab] OR

Pancreatic Intraductal Cancer*[tiab] OR

Pancreas Ductal Carcino* [tiab] OR

Pancreas Ductal Adeno*[tiab] OR

Pancreas Ductal Neoplasm*[tiab] OR

Pancreas Ductal Cancer*[tiab] OR

Pancreas Intraductal Carcino*[tiab] OR

Pancreas Intraductal Adeno*[tiab] OR

Pancreas Intraductal Neoplasm*[tiab] OR

Pancreas Intraductal Cancer*[tiab]

**AND**

"Brachytherapy"[Mesh] OR

Brachytherap*[tiab] OR

Curietherap*[tiab] OR

Intracavit*[tiab] OR

Interstitial*[tiab] OR

intratumo*[tiab] OR

intra-tumo*[tiab] OR

tumour-infiltrat*[tiab] OR

tumourinfiltrat*[tiab] OR

tumour-infiltrat*[tiab] OR

tumourinfiltrat*[tiab] OR

intralesion*[tiab] OR

intra-lesion*[tiab] OR

interstitial*[tiab] OR

implant*[tiab] OR

Local therap*[tiab]

**AND**

unresect*[tiab] OR

non-resect*[tiab] OR

nonresect*[tiab] OR

not-resect*[tiab] OR

unremov*[tiab] OR

LAPC[tiab] OR

Locally advanced pancreatic[tiab] OR

Incompletely resectable*[tiab] OR

incomplete resection*[tiab] OR

Partial-resect*[tiab] OR

Inoper*[tiab] OR

Non-opera*[tiab] OR

Nonopera*[tiab] OR

Not-opera*[tiab] OR

Not-surgic*[tiab] OR

Non-surgic*[tiab] OR

Nonsurgic*[tiab]

## EMBASE

|  | exp pancreas islet cell carcinoma/ or exp pancreas adenoma/ or exp pancreas malformation/ or exp pancreas cancer/ or exp pancreas islet cell tumour/ or exp pancreas adenocarcinoma/ or exp pancreas tumour/ or exp pancreas carcinoma/ or exp pancreas metastasis/ or exp pancreas islet cell hyperplasia/ or exp insulinoma/ or exp gastrinoma/ or exp glucagonoma/ or exp somatostatinoma/ or exp vipoma/ or exp pancreatic cancer cell line/ or exp pancreatic ductal carcinoma cell line/ or |
| --- | --- |

Pancreatic Neoplasm*.ti,ab,kw. OR

Pancreatic adeno*.ti,ab,kw. OR

Pancreas adeno*.ti,ab,kw. OR

Pancreas Neoplasm*.ti,ab,kw. OR

Pancreatic carcino*.ti,ab,kw. OR

Pancreas carcino*.ti,ab,kw. OR

Pancreas tum*.ti,ab,kw. OR

Pancreatic tum*.ti,ab,kw. OR

Cancer of Pancreas.ti,ab,kw. OR

Tumour of the pancreas.ti,ab,kw. OR

Pancreas Cancer*.ti,ab,kw. OR

Pancreatic Cancer*.ti,ab,kw. OR

Cancer of the Pancreas.ti,ab,kw. OR

Carcinoma of the pancreas.ti,ab,kw. OR

Islet Cell Adenoma*.ti,ab,kw. OR

Insulinoma*.ti,ab,kw. OR

Islet Cell Carcinoma*.ti,ab,kw. OR

Gastrinoma*.ti,ab,kw. OR

Glucagonoma*.ti,ab,kw. OR

Somatostatinoma*.ti,ab,kw. OR

Vipoma*.ti,ab,kw. OR

Pancreatic Ductal Carcino*.ti,ab,kw.OR

Pancreatic Ductal Adeno*.ti,ab,kw. OR

Pancreatic Ductal Neoplasm*.ti,ab,kw.OR

Pancreatic Ductal Cancer*.ti,ab,kw. OR

Pancreatic Intraductal Carcino*.ti,ab,kw. OR

Pancreatic Intraductal Adeno*.ti,ab,kw.OR

Pancreatic Intraductal Neoplasm*.ti,ab,kw. OR

Pancreatic Intraductal Cancer*.ti,ab,kw. OR

Pancreas Ductal Carcino*.ti,ab,kw. OR

Pancreas Ductal Adeno*.ti,ab,kw. OR

Pancreas Ductal Neoplasm*.ti,ab,kw. OR

Pancreas Ductal Cancer*.ti,ab,kw. OR

Pancreas Intraductal Carcino*.ti,ab,kw. OR

Pancreas Intraductal Adeno*.ti,ab,kw. OR

Pancreas Intraductal Neoplasm*.ti,ab,kw. OR

Pancreas Intraductal Cancer*:ti,ab,kw

**AND**

exp intratumoral drug administration/ or exp brachytherapy implant/ or exp brachytherapy/ or

Brachytherap*.ti,ab,kw. OR

Curietherap*.ti,ab,kw. OR

Intracavit*.ti,ab,kw. OR

Interstitial*.ti,ab,kw. OR

intratumo*.ti,ab,kw. OR

intra-tumo*.ti,ab,kw. OR

tumour-infiltrat*.ti,ab,kw. OR

tumourinfiltrat*.ti,ab,kw. OR

tumour-infiltrat*.ti,ab,kw. OR

tumourinfiltrat*.ti,ab,kw. OR

intralesion*.ti,ab,kw. OR

intra-lesion*.ti,ab,kw. OR

interstitial*.ti,ab,kw. OR

implant*.ti,ab,kw. OR

Local therap*:ti,ab,kw

**AND**

unresect*.ti,ab,kw. OR

non-resect*.ti,ab,kw. OR

nonresect*.ti,ab,kw. OR

not-resect*.ti,ab,kw. OR

unremov*.ti,ab,kw. OR

LAPC.ti,ab,kw. OR

Locally advanced pancreatic.ti,ab,kw.OR

Incompletely resectable*.ti,ab,kw. OR

incomplete resection*.ti,ab,kw. OR

Partial-resect*.ti,ab,kw. OR

Inoper*.ti,ab,kw. OR

Non-opera*.ti,ab,kw. OR

Nonopera*.ti,ab,kw. OR

Not-opera*.ti,ab,kw. OR

Not-surgic*.ti,ab,kw. OR

Non-surgic*.ti,ab,kw. OR

Nonsurgic*:ti,ab,kw

## Cochrane Library

#1 MeSH descriptor: [Pancreatic Neoplasms] explode all trees

#2

Pancreatic Neoplasm*:ti,ab,kw or

Pancreatic adeno*:ti,ab,kw or

Pancreas adeno*:ti,ab,kw or

Pancreas Neoplasm*:ti,ab,kw or

Pancreatic carcino*:ti,ab,kw or

Pancreas carcino*:ti,ab,kw or

Pancreas tum*:ti,ab,kw or

Pancreatic tum*:ti,ab,kw or

Cancer of Pancreas:ti,ab,kw or

Tumour of the pancreas:ti,ab,kw or

Pancreas Cancer*:ti,ab,kw or

Pancreatic Cancer*:ti,ab,kw or

Cancer of the Pancreas:ti,ab,kw or

Carcinoma of the pancreas:ti,ab,kw or

Islet Cell Adenoma*:ti,ab,kw or

Insulinoma*:ti,ab,kw or

Islet Cell Carcinoma*:ti,ab,kw or

Gastrinoma*:ti,ab,kw or

Glucagonoma*:ti,ab,kw or

Somatostatinoma*:ti,ab,kw or

Vipoma*:ti,ab,kw or

Pancreatic Ductal Carcino*:ti,ab,kw or

Pancreatic Ductal Adeno*:ti,ab,kw or

Pancreatic Ductal Neoplasm*:ti,ab,kw or

Pancreatic Ductal Cancer*:ti,ab,kw or

Pancreatic Intraductal Carcino*:ti,ab,kw or

Pancreatic Intraductal Adeno*:ti,ab,kw or

Pancreatic Intraductal Neoplasm*:ti,ab,kw or

Pancreatic Intraductal Cancer*:ti,ab,kw or

Pancreas Ductal Carcino*:ti,ab,kw or

Pancreas Ductal Adeno*:ti,ab,kw or

Pancreas Ductal Neoplasm*:ti,ab,kw or

Pancreas Ductal Cancer*:ti,ab,kw or

Pancreas Intraductal Carcino*:ti,ab,kw or

Pancreas Intraductal Adeno*:ti,ab,kw or

Pancreas Intraductal Neoplasm*:ti,ab,kw or

Pancreas Intraductal Cancer*:ti,ab,kw

#3 MeSH descriptor: [Brachytherapy] explode all trees

#4

Brachytherap*:ti,ab,kw or

Curietherap*:ti,ab,kw or

Intracavit*:ti,ab,kw or

Interstitial*:ti,ab,kw or

intratumo*:ti,ab,kw or

intra-tumo*:ti,ab,kw or

tumour-infiltrat*:ti,ab,kw or

tumourinfiltrat*:ti,ab,kw or

tumour-infiltrat*:ti,ab,kw or

tumourinfiltrat*:ti,ab,kw or

intralesion*:ti,ab,kw or

intra-lesion*:ti,ab,kw or

interstitial*:ti,ab,kw or

implant*:ti,ab,kw or

Local therap*:ti,ab,kw

#5

unresect*:ti,ab,kw or

non-resect*:ti,ab,kw or

nonresect*:ti,ab,kw or

not-resect*:ti,ab,kw or

unremov*:ti,ab,kw or

LAPC:ti,ab,kw or

Locally advanced pancreatic:ti,ab,kw or

Incompletely resectable*:ti,ab,kw or

incomplete resection*:ti,ab,kw or

Partial-resect*:ti,ab,kw or

Inoper*:ti,ab,kw or

Non-opera*:ti,ab,kw or

Nonopera*:ti,ab,kw or

Not-opera*:ti,ab,kw or

Not-surgic*:ti,ab,kw or

Non-surgic*:ti,ab,kw or

Nonsurgic*:ti,ab,kw

#6 (#1 or #2) and (#3 or #4) and #5

# Appendix S2 – Newcastle Ottawa Quality Assessment Scale

**CASE CONTROL STUDIES**

Note: A study can be awarded a maximum of one star for each numbered item within the Selection and

Exposure categories. A maximum of two stars can be given for Comparability.

**Selection**

1) Is the case definition adequate?

*a) yes, with independent validation* ☆

*b) yes, e.g. record linkage or based on self-reports*

*c) no description*

2) Representativeness of the cases

*a) consecutive or obviously representative series of cases* ☆

*b) potential for selection biases or not stated*

3) Selection of Controls

*a) community controls* ☆

*b) hospital controls*

*c) no description*

4) Definition of Controls

*a) no history of disease (endpoint)* ☆

*b) no description of source*

**Comparability**

1) Comparability of cases and controls on the basis of the design or analysis

*a) study controls for cancer staging* ☆

*b) study controls for (neo)adjuvant therapy* ☆

**Exposure**

1) Ascertainment of exposure

*a) secure record (e.g. surgical records)* ☆

*b) structured interview where blind to case/control status* ☆

*c) interview not blinded to case/control status*

*d) written self-report or medical record only*

*e) no description*

2) Same method of ascertainment for cases and controls

*a) yes* ☆

*b) no*

3) Non-Response rate

*a) same rate for both groups* ☆

*b) non respondents described*

*c) rate different and no designation*

**COHORT STUDIES**

Note: A study can be awarded a maximum of one star for each numbered item within the Selection and

Outcome categories. A maximum of two stars can be given for Comparability

**Selection**

*1) Representativeness of the exposed cohort*

*a) truly representative of the average patient with locally advanced pancreatic cancer in the community* ☆

*b) somewhat representative of the average patient with locally advanced pancreatic cancer in the community* ☆

*c) selected group of users e.g. nurses, volunteers*

*d) no description of the derivation of the cohort*

2) Selection of the non-exposed cohort

*a) drawn from the same community as the exposed cohort* ☆

*b) drawn from a different source*

*c) no description of the derivation of the non-exposed cohort*

3) Ascertainment of exposure

*a) secure record (e.g. surgical records)* ☆

*b) structured interview* ☆

*c) written self-report*

*d) no description*

4) Demonstration that outcome of interest was not present at start of study

*a) yes* ☆

*b) no*

**Comparability**

*1) Comparability of cohorts on the basis of the design or analysis*

*a) study controls for cancer staging* ☆

*b) study controls for (neo)adjuvant therapy* ☆

**Outcome**

*1) Assessment of outcome*

*a) independent blind assessment* ☆

*b) record linkage* ☆

*c) self-report*

*d) no description*

2) Was follow-up long enough for outcomes to occur

*a) yes (select an adequate follow up period for outcome of interest)* ☆

*b) no*

3) Adequacy of follow up of cohorts

*a) complete follow up - all subjects accounted for* ☆

*b) subjects lost to follow up unlikely to introduce bias - small number lost - >90 % follow up, or description provided of those lost* ☆

*c) follow up rate < 90% and no description of those lost*

*d) no statement*

# Appendix S3 – Newcastle Ottawa Quality Assessment Scale Results

## Quality assessment of the cohort studies by the Newcastle-Ottawa Scale

| **Study** | **Selection** | **Comparability** | **Outcome of Interest** |
| --- | --- | --- | --- |
| Bhutani, M.S. et al. | ☆ | ☆☆ | ☆ |
| Dobelbower, R.R. Jr. et al. | ☆☆☆ | ☆☆ | ☆☆☆ |
| Du, Y. et al. | ☆☆☆ | ☆☆ | ☆☆☆ |
| Gong, T. et al. | ☆☆☆ | ☆ | ☆☆☆ |
| Hecht, J.R. et al. | ☆☆ | - | ☆☆ |
| Hecht, J.R. et al. | ☆☆☆ | ☆☆ | ☆☆☆ |
| Herman, J.M. et al.* | ☆☆☆☆ | ☆ | ☆☆☆ |
| Hirooka, Y. et al. | ☆☆☆ | ☆☆ | ☆☆☆ |
| Jin, Z. et al. | ☆☆☆ | - | ☆☆☆ |
| Joyce, F. et al. | ☆☆☆ | ☆☆ | ☆☆☆ |
| Levy, M.J. et al. | ☆☆ | - | ☆☆☆ |
| Li, J.L. et al. | ☆☆☆ | ☆ | ☆☆☆ |
| Li, J.Q. et al.* | ☆☆☆ | ☆ | ☆☆☆ |
| Lun, J.J. et al. | ☆☆ | - | ☆☆ |
| Mohamadnejad, M. et al. | ☆☆☆ | ☆☆ | ☆☆☆ |
| Montemaggi, P. et al. | ☆☆ | - | ☆☆☆ |
| Mutignani, M. et al. | ☆☆☆ | - | ☆☆☆ |
| Nishimura, M. et al. | ☆☆ | - | ☆☆☆ |
| Niu, L. et al. | ☆☆☆ | ☆ | ☆☆ |
| Nori D, et al. | ☆☆ | ☆ | ☆☆ |
| Order, S.E. et al. | ☆☆☆ | ☆☆ | ☆☆☆ |
| Raben, A. et al. | ☆☆☆ | ☆ | ☆☆☆ |
| Rosemurgy, A. et al.* | ☆☆☆☆ | ☆ | ☆☆☆ |
| Ross, P.J. et al. | ☆☆☆ | - | ☆☆ |
| Shipley, W.U. et al. | ☆☆☆ | ☆ | ☆☆ |
| Sun, S. et al. | ☆☆☆ | ☆☆ | ☆☆☆ |
| Syed, A.M. et al. | ☆☆☆ | ☆ | ☆☆☆ |
| Wang, B. | ☆☆☆ | - | ☆☆☆ |
| Wang, H. et al. | ☆☆ | - | ☆☆ |
| Wang, J. et al. | ☆ | ☆ | ☆☆ |
| Wang, W. et al. | ☆☆☆ | ☆ | ☆☆ |
| Westlin, J.E. et al. | ☆☆☆ | - | ☆☆☆ |
| Xiao, B. et al.* | ☆☆☆ | ☆ | ☆☆ |
| Xu, K.C. et al. | ☆☆☆ | - | ☆☆☆ |
| Yang, B. et al. | ☆☆☆ | ☆ | ☆☆☆ |
| Yang, M. et al. | ☆☆ | - | ☆☆☆ |
| Yunwei, S. et al. | - | - | ☆☆☆ |
| Zheng, Z. et al. | ☆☆☆☆ | - | ☆☆☆ |
| Zhongmin, W. et al. | ☆☆☆ | ☆ | ☆☆☆ |
| Zou, Y.P. et al. | ☆☆☆ | ☆☆ | ☆☆ |

## Quality assessment of the case-control studies by the Newcastle-Ottawa Scale

| **Study** | **Selection** | **Comparability** | **Exposure** |
| --- | --- | --- | --- |
| Goertz, S.R. et al. | ☆☆☆☆ | ☆ | ☆☆ |
| Li, W. et al. | ☆☆☆☆ | ☆ | ☆☆☆ |
| Li, Y.F. et al. | ☆☆☆☆ | ☆☆ | ☆☆☆ |
| Liu, K. et al. | ☆☆☆ | ☆☆ | ☆☆☆ |
| Luo, M. et al. | ☆☆☆☆ | - | ☆☆☆ |
| Mohiuddin, M. et al. | - | ☆ | ☆☆ |
| Morrow, M. et al. | ☆☆ | - | ☆☆ |
| Peretz, T. et al. | ☆☆ | ☆ | ☆ |
| Schad, F. et al. | - | ☆☆ | ☆ |
| Schuricht, A. L. et al. | ☆☆☆ | ☆☆ | ☆☆☆ |
| Sun, X. et al. | - | ☆ | ☆ |
| Whittington, R. et al. | ☆☆☆☆ | ☆ | ☆☆☆ |

Appendix S4 – An overview of applied radioactive isotopes for intratumoral injection therapies in pancreatic cancer

| Isotope | Decay | T_1/2_ (days) | Mean energy (KeV) | Mean tissue penetration (mm) | Mean particle size |
| --- | --- | --- | --- | --- | --- |
| Iodine-125 | Electron capture | 59.49 | 27.5 | 17 | 4.4 - 4.6 mm |
| Phosphorus-32 | Beta-minus | 14.29 | 695 | 2 - 3 | 20 nm - 30 µm |
| Palladium-103 | Electron capture | 16.99 | 20.2 | <17 | 4.5 - 4.7 mm |
